# Microbial community composition of transiently wetted Antarctic Dry Valley soils

**DOI:** 10.3389/fmicb.2015.00009

**Published:** 2015-01-28

**Authors:** Thomas D. Niederberger, Jill A. Sohm, Troy E. Gunderson, Alexander E. Parker, Joëlle Tirindelli, Douglas G. Capone, Edward J. Carpenter, Stephen C. Cary

**Affiliations:** ^1^College of Marine and Earth Sciences, University of DelawareLewes, DE, USA; ^2^Wrigley Institute for Environmental Studies and Department of Biological Sciences, University of Southern CaliforniaLos Angeles, CA, USA; ^3^Romberg Tiburon Center for Environmental Studies, San Francisco State UniversityTiburon, CA, USA; ^4^School of Science, University of WaikatoHamilton, New Zealand

**Keywords:** Dry, Valley, Antarctica, microbial diversity, microbial community, hyporheic

## Abstract

During the summer months, wet (hyporheic) soils associated with ephemeral streams and lake edges in the Antarctic Dry Valleys (DVs) become hotspots of biological activity and are hypothesized to be an important source of carbon and nitrogen for arid DV soils. Recent research in the DV has focused on the geochemistry and microbial ecology of lakes and arid soils, with substantially less information being available on hyporheic soils. Here, we determined the unique properties of hyporheic microbial communities, resolved their relationship to environmental parameters and compared them to archetypal arid DV soils. Generally, pH increased and chlorophyll *a* concentrations decreased along transects from wet to arid soils (9.0 to ~7.0 for pH and ~0.8 to ~5 μg/cm^3^ for chlorophyll *a*, respectively). Soil water content decreased to below ~3% in the arid soils. Community fingerprinting-based principle component analyses revealed that bacterial communities formed distinct clusters specific to arid and wet soils; however, eukaryotic communities that clustered together did not have similar soil moisture content nor did they group together based on sampling location. Collectively, rRNA pyrosequencing indicated a considerably higher abundance of Cyanobacteria in wet soils and a higher abundance of Acidobacterial, Actinobacterial, *Deinococcus/Thermus*, Bacteroidetes, Firmicutes, Gemmatimonadetes, Nitrospira, and Planctomycetes in arid soils. The two most significant differences at the genus level were *Gillisia* signatures present in arid soils and chloroplast signatures related to *Streptophyta* that were common in wet soils. Fungal dominance was observed in arid soils and Viridiplantae were more common in wet soils. This research represents an in-depth characterization of microbial communities inhabiting wet DV soils. Results indicate that the repeated wetting of hyporheic zones has a profound impact on the bacterial and eukaryotic communities inhabiting in these areas.

## INTRODUCTION

The Antarctic continent is one of the most physically and chemically demanding environments on Earth and was once considered to be adverse to all life (Boyd et al., 1966). The extreme nature of this environment is exemplified in the Dry Valleys (DVs) of South Victoria Land, Eastern Antarctica, which comprise the largest contiguous ice-free region in Antarctica, with glaciated valley systems covering an area over 6000 km^2^ (Cary et al., 2010). Antarctic DV soil ecosystems are characterized by huge temporal variations in temperature and light regimes (Thompson et al., 1971; Vincent, 1988; Doran et al., 2002), steep chemical gradients, and a high incidence of solar radiation in austral summer (Smith et al., 1992; Dana et al., 1998). The microbes inhabiting these ice-free cold soil environments are subjected to extremely low nutrients (Vishniac, 1993) and low bioavailability of water with by high levels of salinity (Claridge and Campbell, 1977; Bockheim, 1997; Barrett et al., 2004). The additive effects of such extreme aridity, and widely fluctuating physiochemical conditions greatly impacts the adaptations and life cycle strategies used by the resident biota in these “cold deserts.”

The biota within the DV mineral soils is comprised of bacteria, microalgae, fungi, and microbial feeding heterotrophs such as nematodes, rotifers, and tardigrades (Wynn-Williams, 1996; Moorhead et al., 2003); however, prokaryotes represent the greatest biomass in Antarctic ecosystems (Franzmann, 1996). Isolation and description of the microflora from the continent over the last six decades has yielded numerous novel microbial species (Johnson et al., 1978; Vishniac, 1993; de la Torre et al., 2003), but much remains unknown about the composition and ecosystem functioning of these microbial communities.

Early culture-based microbial studies (Cameron et al., 1970, Vishniac and Mainzer, 1972; Vincent, 1988; Wynn-Williams, 1990; Friedmann, 1993; Vishniac, 1993) suggested that the soil bacterial diversity and abundance in these cold desert areas was extremely low, as might be predicted by the extreme nature of the system. This is in stark contrast to subsequent work in nutrient- and water-rich Antarctic ‘ornithogenic’ and fell-field soils, and lake sediments showing vastly higher microbial abundance based on direct counts (Ramsay and Stannard, 1986; Wynn-Williams, 1990; Sjoling and Cowan, 2003). However, not until recent molecular-based phylogenetic studies on DV microbial populations (de la Torre et al., 2003; Smith et al., 2006; Niederberger et al., 2008) was the true, and surprisingly high diversity of microorganisms in this environment revealed (Cary et al., 2010). This was an unexpected finding considering the extreme nature and trophic simplicity of the system.

While considerable research has focused on the geochemistry and microbial ecology of the permanently ice covered lakes (Priscu et al., 1998; Gordon et al., 2000; Berry Lyons et al., 2001; Berry Lyons, 2004) and the arid DV soils (Smith et al., 2006, 2010; Wood et al., 2008; Babalola et al., 2009; Pointing et al., 2009; Lee et al., 2012) there is substantially less information on the ecology and biogeochemistry of the streams feeding these lakes and the wet moated areas surrounding lakes (Stanish et al., 2013). DV lakes are fed by transient glacial melt streams that typically flow for 6–12 weeks during the austral summer and are virtually the only source of water for the DV (Gooseff et al., 2006). The wet zone of soil which extends below and to the sides of glacial streams (and lake edges), defined as the hyporheic zone, can be up to about 70 cm deep (to the permafrost depth) and extend several meters to each side (Gooseff et al., 2006). A feature of many of the hyporheic zones is the conspicuous presence of cyanobacteria in biological crust communities. These communities vary greatly in the abundance and types of dominant cyanobacteria and can vary from one stream to another and from one valley to another (Broady, 1982; Vincent and Howard-Williams, 1986; Vincent et al., 1993; McKnight and Tate, 1997; Vincent, 2000). During the austral winter these communities are essentially freeze-dried and inactive. Various studies have demonstrated that during the warmer summer months DV hyporheic soils are hotspots of biological cycling (Runkel et al., 1998; McKnight et al., 1999, 2007; Gooseff et al., 2003), undertake high levels of N_2_ fixation (Howard-Williams et al., 1989; Niederberger et al., 2012) and are thought to be an important source of organic C and N to the surrounding soils due to aeolian-associated distribution of crust detritus (Hopkins et al., 2006a,b; Barrett et al., 2007; Novis et al., 2007). As a result, primary productivity and N_2_ fixation at these wet sites may represent a major source of C and N to an otherwise nutrient-limited system. Although the significance of these transiently wet sites is clear, there is little information on the microbial assemblages inhabiting these soils. Recent work on sediments in streams shows specific diatoms and bacteria co-occur (Stanish et al., 2013), but our study presented here provides the first comprehensive view of microbial communities (prokaryotic and eukaryotic) in the wet regions adjacent to streams and lakes as compared to arid soils in the DV. Therefore, the objective of this study was to add to this knowledge gap and characterize and compare microbial communities of both wet (hyporheic) soil zones and local bulk arid soils of the Antarctic DV. Detailed rRNA gene-based analyses was used to shed light on the major organisms inhabiting these soils and indicate that microbial communities differ considerably between these contrasting biotopes.

## MATERIALS AND METHODS

### COLLECTION AND SOIL ANALYSES

**FIGURE 1:**
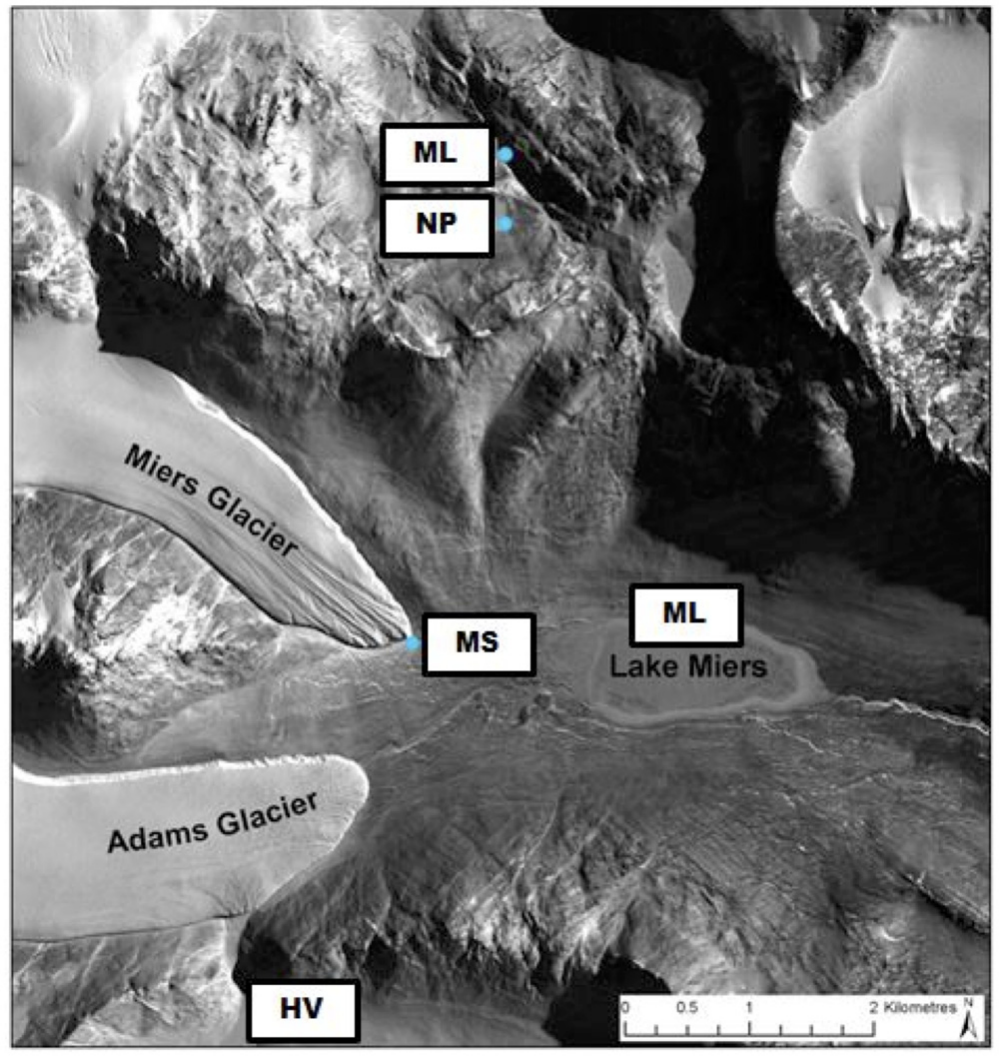
**Location of sampling sites**.

Sampling sites and methodology is as described previously (Niederberger et al., 2012). Transects ranging between 3.7 and 20 m in length were sampled that consisted of four sampling sites originating from site 1 defined as a ‘wet’ zone (sampling within the lake/stream edge) extending through moist soil zones containing crust communities (sites 2 and 3) to the final site 4 located in a typical DV desert mineral soil. A total of 10 transects were sampled during two separate field seasons (January and December, 2009) as listed in **Table 1**. Transects collected during the initial field season were also described in a previous study describing N_2_ fixation activities in the wet soils (Niederberger et al., 2012). The locations of the sampled transects are presented in **Figure 1** and included: the northern shore of Miers Lake (ML) in Miers Valley; sites both at the source (Miers Glacier) and downstream of Miers Stream (MS) which feeds into ML; a pond situated over the southern ridge of Miers Valley [Hidden Valley (HV)]; and two lake systems [Nostoc Pond (NP) and Buddha Lake (BL)] located over the north ridge of Miers Valley. Soil pH, gravimetric water content, chlorophyll *a*, nitrate and nitrite, ammonium, silicate, orthophosphate, and cell counts were determined as described previously (Niederberger et al., 2012). Nutrient concentrations were not determined for some of the highly arid soils due to the lack of pore water.

**Table 1:** Sampled transects and associated metadata.

Sample	Location	Collection (2009)	GPS	Distance from wet source (m)	Water content (%)	pH	*Chl a* per cm^3^ soil (μg)	NO_3_+NO_2_(μM)	NH_4_(μM)	SiOH_4_ (μM)	PO_4_(μM)
MS1-1	Miers Stream	18th January	S78° 05.615’; E163°49.912’	Origin	Submerged	7.01	0.0129	44.16	2.15	90.07	0.35


MS1-2				9.6	4.5	9.26	5.0438	39.42	2.61	76.56	0.29
MS1-3				13.5	19.2	8.58	0.0598	36.86	1.01	52.41	0.51
MS1-4				20.0	3.2	9.94	0.3284	22.88	1.72	151.35	2.47

MS2-1	Miers Stream	22nd January	S78°05.745’; E163°45.885’	Origin	17.78	8.05	0.0948	3.95	0.78	0.95	0.17


MS2-2				3.0	5.84	7.87	0.1645	nd	nd	nd	nd
MS2-3				4.5	2.29	8.33	0.1540	nd	nd	nd	nd
MS2-4				6.5	0.27	8.35	0.0158	nd*	nd*	nd*	nd*

ML1-1	Miers Lake (North)	19th January	S78° 05.615’; E163°49.912’	Origin	Submerged	8.59	0.4857	25.73	1.38	63.71	0.79


ML1-2				3.0	22.73	7.70	0.1020	54.1	1.26	130.87	1.87
ML1-3				3.7	14.54	9.52	0.2018	17.81	1.34	184.78	2.17
ML1-4				8.3	2.10	9.02	0.0104	nd*	nd*	nd*	nd*

NP1	Nostoc Pond	20th January	S78° 03.920’; E163°46.497’	Origin	32.74	7.63	0.0339	19.2	2.02	170.52	0.42


NP2				0.03	31.35	7.19	0.0608	25.54	1.15	213.82	0.87
NP3				1.8	11.43	8.35	0.2586	29.99	3.39	256.34	0.56
NP4				3.7	3.29	8.86	nd	nd*	nd*	nd*	nd*

BL1	Buddha Lake	24th January	S78°03.621’; E163°46.463’	Origin	34.14	7.48	0.0526	0.54	1.94	62.39	0.17


BL2				1.0	22.75	7.66	0.1636	0.43	81.39	154.24	0.68
BL3				2.0	17.92	7.92	0.2176	0.44	66	162.32	0.24
BL4				6.0	0.35	9.20	0.2324	0.52	51.92	100.17	0.17

MLN-1^2^	Miers Lake (North)	13th December	E163°49.908’ S78°05.613’;	Origin	24.12	7.95	2.138	0	2.05	19.49	0


MLN-2^2^				2.3	17.59	8.22	0.115	11.25	1.47	142.46	0.75
MLN-3^2^				3.4	23.87	9.31	4.488	14.11	1.65	153.21	1.86
MLN-4^2^				9.0	0.35	8.23	0.119	nd*	nd*	nd*	nd*

NP1^2^	Nostoc Pond	08th December	S78°03.927’; E163°46.310’	Origin	Submerged	7.55	0.07	0	11.85	0	0.32


NP2^2^				0.5	49.51	7.58	0.212	1.3	4.66	163.83	2.95
NP3^2^				1.3	29.06	7.68	0.593	30.35	3.58	184.29	0.66
NP4^2^				10.0	0.26	8.33	0.159	nd*	nd*	nd*	nd*

HV1^2^	Hidden Valley Lake	14th December	S78°07.361’; E163°41.544’	Origin	56.77	8.74	2.841	0	1.23	113.01	0


HV2^2^				0.7	41.65	8.83	3.466	0.13	12.42	178.33	1.13
HV3^2^				4.7	10.37	9.00	6.117	0.33	1.20	92.28	0.31

MS1-1^2^	Miers Stream	12th December	S78°05.955’; E163°44.834’	Origin	11.91	8.91	1.386	nd	nd	nd	nd


MS1-2^2^				4.0	34.02	8.36	0.104	3.81	1.88	16.3	0
MS1-3^2^				15.0	0.09	9.71	0.112	nd*	nd*	nd*	nd*

MS2-1^2^	Miers Stream	16th December	S78°06.091’; E163°49.364’	Origin	27.00	7.77	1.852	0	1.07	19.93	0.13


MS2-2^2^				1.4	30.64	7.91	0.508	27.69	3.67	101.14	0.88
MS2-3^2^				2.5	37.99	8.72	4.003	5.96	2.70	95.42	1.19
MS2-4^2^				6.8	0.73	9.16	0.882	nd*	nd*	nd*	nd*

The initial five transects were collected during the first field season (January, 2009) and the remainder during the second season (December, 2009). nd, not determined; nd*, a lack of sufficient pore water for nutrient analysis.

### DNA EXTRACTION AND TERMINAL-RESTRICTION FRAGMENT LENGTH POLYMORPHISM (T-RFLP) ANALYSES

DNA was isolated from homogenized soil sampled soils using the Powersoil^TM^ DNA isolation kit (MOBIO) as per manufacturer’s instructions with all terminal-restriction fragment length polymorphisms (T-RFLPs) based on pooled triplicate PCR assays (Niederberger et al., 2012). Bacterial T-RFLP analyses were undertaken according to Danovaro et al. (2006). Eukaryotic T-RFLP was performed as described by Díez et al. (2001) with the exception of using a single restriction endonuclease (MspI; New England BioLabs Inc.) as previously performed on Antarctic soils (Pointing et al., 2009). Digestions comprised 3 h incubation at 37°C with an enzyme inactivation step of 80°C for 20 min. Terminal-fragments were sized using the MegaBACE system (Amersham) at the Waikato DNA Sequencing Facility (University of Waikato, Hamilton, New Zealand). Fluorescent peak data were aligned using the *T-REX* online platform (Culman et al., 2009) and the resulting TRF absence/presence data matrix imported into PRIMER6 (Primer-E Ltd., Plymouth, UK) for statistical analyses. Community comparisons were performed via principle component analyses (PCAs) with percentage similarity between community fingerprints overlaid as detailed in the Primer v6: User Manual/Tutorial (Clarke and Gorley, 2006), and BEST analyses used to reveal any abiotic factors that best explain the observed community fingerprints (Clarke and Gorley, 2006).

### AMPLICON PYROSEQUENCING AND ASSOCIATED ANALYSES

Tagged amplicon pyrosequencing of the V1 to V3 region of the 16S and 18S rRNA gene (primer pairs 28F-519R and euk7F-euk570R, respectively) was performed on DNA extracts by the Research and Testing Laboratories located in Lubbock, TX (https://www.researchandtesting.com) using FLX technology (Roche). The pyrosequencing datasets generated in this study are deposited in the European Nucleotide Archive, under accession number PRJEB7939 (ERP008940). Sequence data were quality trimmed (>200 bp) and processed using defaults within the Quantitative Insights Into Microbial Ecology (QIIME) toolkit (Caporaso et al., 2010). In brief, sequences were split according to tags and binned into operational taxonomic units (OTUs) at 95% similarity. Bacterial taxonomic assignment was undertaken on all quality trimmed sequences using the online Ribosomal Database Project (RDP) classifier tool [at 80% confidence level (Cole et al., 2009)]. Eukaryotic taxonomic assignment was performed within the QIIME toolkit using the Basic Local Alignment Search Tool (BLAST) upon a representative sequence from each OTU against the modified SILVA 18S rRNA gene database (Pruesse et al., 2007) as obtained from mothur (Schloss et al., 2009). The online RDP Library compare tool (Cole et al., 2009) was used to evaluate any significant differences in bacterial communities between combined wet and dry soil sequence libraries, with 9039 sequences randomly removed from the NP2 library to ensure the number of sequences remained below the maximum limit of 40,000.

## RESULTS

Samples were collected along transects extending through wet/hyporheic soils from a wet source represented by a lake or stream to typical bulk arid DV soil. Water content in these soils generally decreased along the transects to below ~3% in the arid soils, with a concurrent increase in the pH of the soils from submerged to arid soils (**Table 1**). Chlorophyll *a* concentrations were highest in wet soils (up to ~5 μg/cm^3^) with no obvious correlations between nutrient concentrations and distance from the water source.

**FIGURE 2:**
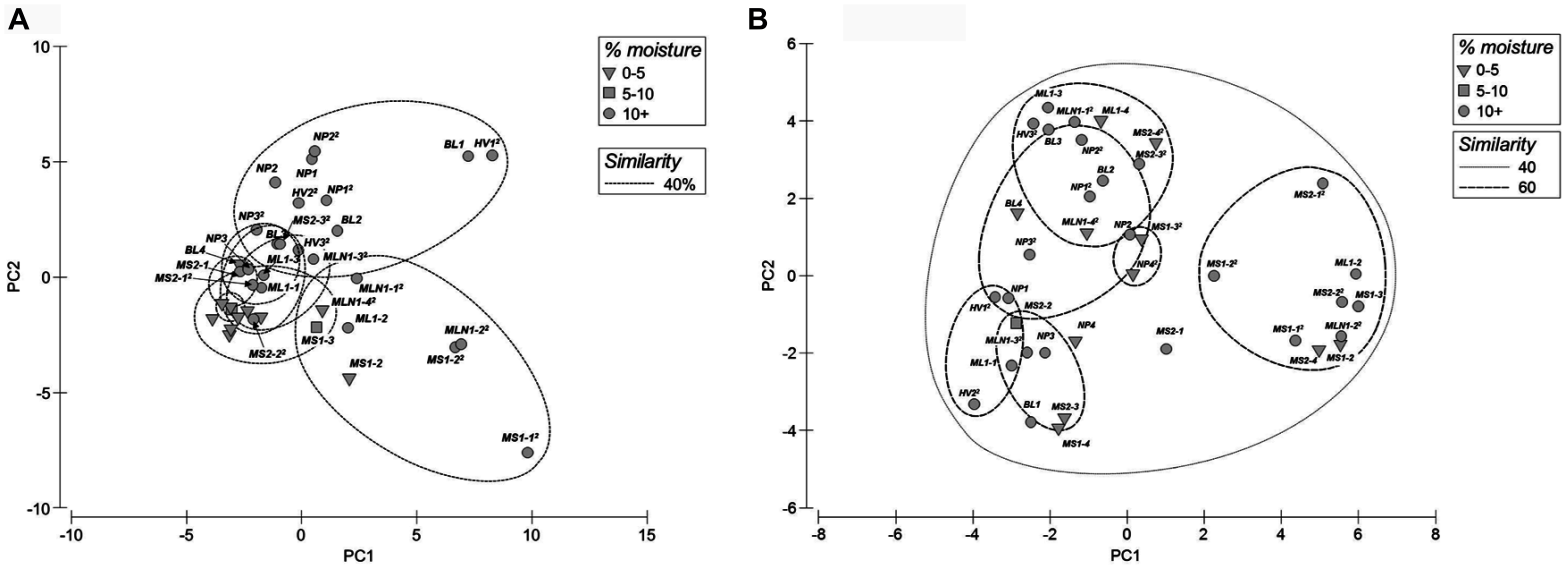
**Principal component analyses of T-RFLP fingerprints from both bacterial **(A)** and eukaryotic **(B)** communities (MS1-1 not included in plots)**.

Principle component analyses of bacterial T-RFLP fingerprints revealed that the arid soils (0–5% water moisture) formed a tight cluster grouping at 40% similarity (**Figure 2A**). Whereas, in general, the wetter sites (>5% moisture content) formed two major clusters grouping at 40% similarity, with one cluster comprised of sites located in Miers Valley (i.e., stream and lake systems) and a second cluster consisting of transects located in both the northern (NP and BL) and southern sites (Hidden Valley). In contrast to the bacterial profiles, high spatial variability was observed for the eukaryotic T-RFLP-based PCA profiles (**Figure 2B**). BEST analysis showed that only a small amount of community structure could be attributed to the environmental parameters measured, bacterial community structure best correlated to distance from water source, pH and NH_4_ (ϱ = 0.214), and the eukaryote community to distance from water source, pH and % moisture (ϱ = 0.202).

**FIGURE 3:**
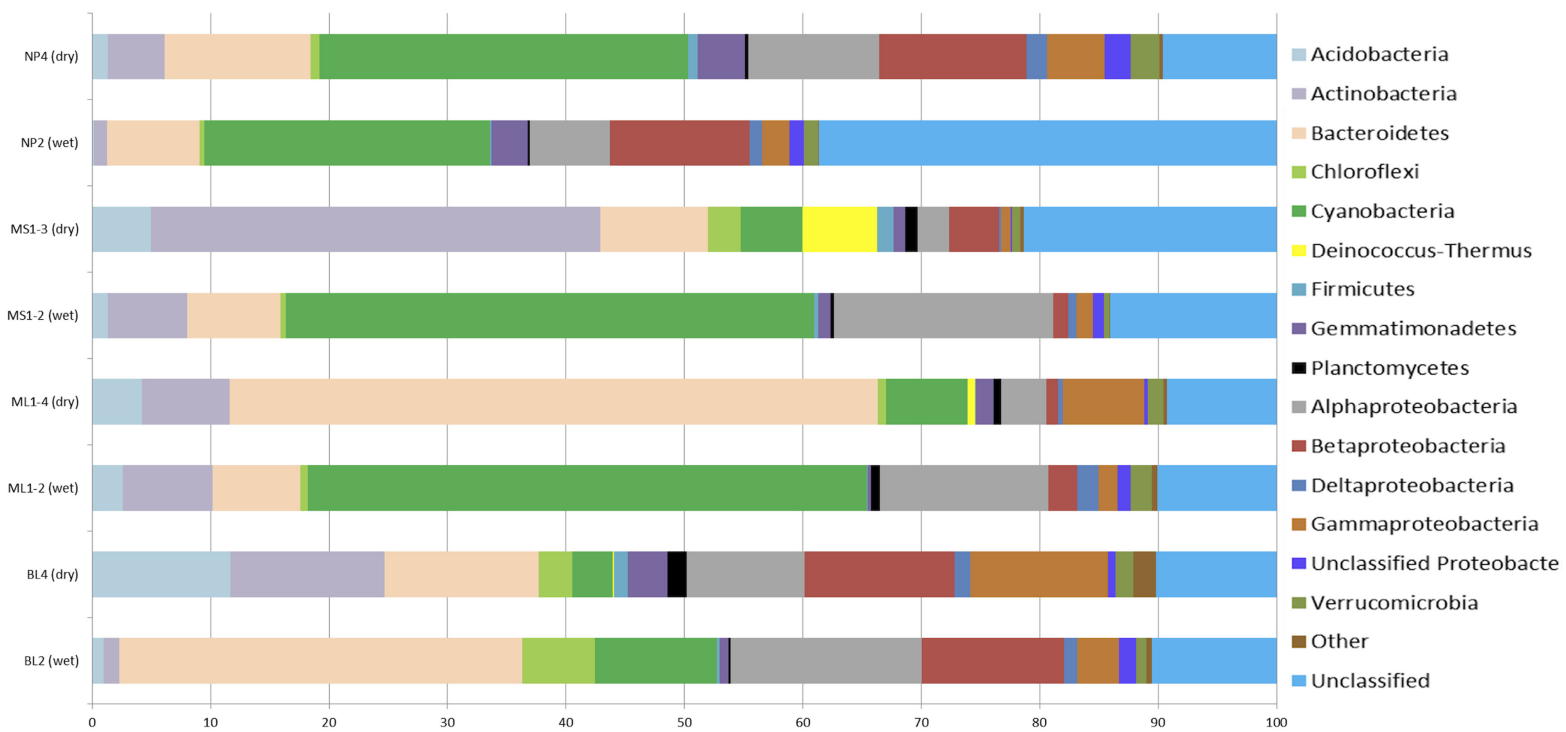
**Percentage composition of bacterial communities as based 16S rRNA-based amplicon pyrosequencing.** The category ‘Other’ includes the phyla: BRC1, OD1, TM7, OP10, Nitrospira, SR1, and WS3. The total number of sequences for each sample: NP4, 7236; NP2, 24000; MS1-3, 5142; MS1-2, 10366; ML1-4, 7163; ML1-2, 7640; BL4, 9957; BL2, 7033.

To further elucidate differences in microbial community structure between wet and arid soils, amplicon pyrosequencing of bacterial and eukaryotic rRNA genes was undertaken on wet and arid soil samples from representative transects collected in January 2009 (transects: BL, ML1) and December 2009 (transects: MS1 and NP). These sites were selected based on clustering PCA results (i.e., wet vs. dry sites were distinctly separated in PCA results) and it was ensured that both a wet and arid site from the same transect was sequenced. Rarefaction analyses at 95% for all sites indicate adequate sampling size with curves reaching or nearing plateau (results not shown). Obvious differences in bacterial composition between wet and dry soil biotopes include increased representation of Acidobacteria and Actinobacteria in arid soils and, with the exception of NP, cyanobacteria being more highly represented in wetter soils (**Figure 3**). Specifically, Acidobacteria comprised ~<2.5% of the total signatures detected in wet soils and 1.3–11.0% in arid soils while Actinobacteria comprised ~<7.5% in wet soils and ~4.7–37.0% in arid soils. As expected, Cyanobacteria comprised ~10–47% of the total sequences in wet soils that had visible microbial crusts and <7% for arid soils of BL, ML, MS (~33% for NP). Of note, the *Deinococcus-Thermus* group was generally present in arid soils (0.09–6.0%), but very low to undetected in the wet soils (<0.02%).

To elucidate overall shared differences of the bacterial rRNA signatures between wet and arid soils, sequences from all wet sites (i.e., BL2, ML1.2, MS1.2, and NP2) were combined and compared to combined arid sites (i.e., BL4, ML1.4, MS1.3, and NP4) using the RDP Library compare tool. Nine phyla and 36 genera showed highly significant differences between wet and arid soils (**Table 2**). Differences in bacterial phyla composition observed when comparing wet and arid soils within each transect (i.e., Acidobacteria, Actinobacteria, Cyanobacteria and *Deinococcus-Thermus*, **Figure 3**) are supported by the combined analysis which also shows that Bacteroidetes, Firmicutes, Gemmatimonadetes, Nitrospira, and Planctomycetes were significantly more abundant in arid samples (**Table 2**). The most significant differences at the genus level was the detection of *Gillisia* signatures in arid soils (~3.7% of the combined sequences) but undetected in wet soils and the chloroplast *Streptophyta-*related signature common in wet soils (~12.0% of combined sequences) while only a single sequence detected in arid soils. See **Table 2** for the list of the most significant results (*e* values ≤ 6.00E^-14^).

**Table 2:** The most significant results (*e* values ≤ 6.00E-14) at the genus level from the comparison of the combined wet soil-associated bacterial sequences (39,999 sequences from BL2, ML1-2, MS1-2, and NP2) with combined arid-associated sequences (29,498 sequences from BL4, ML1-4, MS1-3, and NP4) using the online RDP library compare tool.

Genus	Phylum (Class for Proteobacteria)	Family	Arid soils: # of sequences	Wet soils: # of sequences	Significance
**Phylum level**
*N.A.*	*Deinococcus-Thermus*	*N.A.*	373 (1.3%)	3 (<0.1%)	2.18E-133
*N.A.*	*Acidobacteria*	*N.A.*	1818 (6.2%)	418 (1.0%)	6.00E-14
*N.A.*	*Actinobacteria*	*N.A.*	4116 (14%)	1549 (3.9%)	6.00E-14
*N.A.*	*Bacteroidetes*	*N.A.*	6569 (22.3%)	4865 (12.2%)	6.00E-14
*N.A.*	*Firmicutes*	*N.A.*	246 (0.8%)	67 (0.2%)	6.00E-14
*N.A.*	*Gemmatimonadetes*	*N.A.*	776 (2.6%)	608 (1.5%)	6.00E-14
*N.A.*	*Nitrospira*	*N.A.*	110 (0.4%)	10 (<0.1%)	6.00E-14
*N.A.*	*Planctomycetes*	*N.A.*	286 (1.0%)	134 (0.3%)	6.00E-14
*N.A.*	*Cyanobacteria*	*N.A.*	3351 (11.4%)	13354 (33.4%)	6.00E-14
**Genus level**
* Gillisia*	*Bacteroidetes*	*Flavobacteriaceae*	1092 (3.7%)	0	0.00E+00
*Streptophyta*	*Cyanobacteria*	*Chloroplast*	1 (<0.1%)	4787 (12.0%)	0.00E+00
*Patulibacter*	*Actinobacteria*	*Patulibacteraceae*	399 (1.4%)	5 (<0.1%)	1.53E-139
*Truepera*	*Deinococcus-Thermus*	*Trueperaceae*	357 (1.2%)	3 (<0.1%)	1.72E-127
*Rubrobacter*	*Actinobacteria*	*Rubrobacteraceae*	215 (0.7%)	4 (<0.1%)	8.62E-74
*Sphaerobacter*	*Chloroflexi*	*Sphaerobacteraceae*	168 (0.6%)	4 (<0.1%)	1.02E-56
*Arthrobacter*	*Actinobacteria*	*Micrococcaceae*	120 (<0.1%)	3 (<0.1%)	1.12E-40
*Rheinheimera*	*Gammaproteobacteria*	*Chromatiaceae*	106 (<0.1%)	0	3.01E-40
*Nitriliruptor*	*Actinobacteria*	*Nitriliruptoraceae*	104 (<0.1%)	1 (<0.1%)	1.03E-37
*Pseudomonas*	*Gammaproteobacteria*	*Pseudomonadaceae*	87 (<0.1%)	1 (<0.1%)	1.83E-31
*Flavisolibacter*	*Bacteroidetes*	*Chitinophagaceae*	71 (<0.1%)	2 (<0.1%)	2.92E-24
*Anoxybacillus*	*Firmicutes*	*Bacillaceae*	52 (<0.1%)	0	3.77E-20
*Erythromicrobium*	*Alphaproteobacteria*	*Erythrobacteraceae*	0	66 (0.2%)	1.68E-16
*Paenisporosarcina*	*Firmicutes*	*Planococcaceae*	37 (<0.1%)	0	1.44E-14
*Algoriphagus*	*Bacteroidetes*	*Cyclobacteriaceae*	94 (<0.1%)	11 (<0.1%)	6.00E-14
*Amaricoccus*	*Alphaproteobacteria*	*Rhodobacteraceae*	6 (<0.1%)	276 (0.7%)	6.00E-14
*Brevundimonas*	*Alphaproteobacteria*	*Caulobacteraceae*	193 (0.7%)	737 (1.8%)	6.00E-14
*Caldilinea*	*Chloroflexi*	*Caldilineaceae*	153 (0.5%)	66 (0.2%)	6.00E-14
*Duganella*	*Betaproteobacteria*	*Oxalobacteraceae*	163 (0.6%)	28 (<0.1%)	6.00E-14
*Flavobacterium*	*Bacteroidetes*	*Flavobacteriaceae*	681 (2.3%)	2083 (5.2%)	6.00E-14
*Gemmatimonas*	*Gemmatimonadetes*	*Gemmatimonadaceae*	776 (2.6%)	608 (1.5%)	6.00E-14
*Gp16*	*Acidobacteria*	*N.A.*	355 (3.4%)	74 (0.2%)	6.00E-14
*Gp4*	*Acidobacteria*	*N.A.*	994 (1.2%)	251 (0.6%)	6.00E-14
*Gp6*	*Acidobacteria*	*N.A.*	348 (1.2%)	56 (0.1%)	6.00E-14
*Gp7*	*Acidobacteria*	*N.A.*	88 (<0.1%)	15 (<0.1%)	6.00E-14
*GpI*	*Cyanobacteria*	*Family I*	320 (1.1%)	2514 (6.3%)	6.00E-14
*GpIV*	*Cyanobacteria*	*Family IV*	563 (1.9%)	461 (1.2%)	6.00E-14
*Iamia*	*Actinobacteria*	*Iamiaceae*	183 (0.6%)	35 (0.1%)	6.00E-14
*Lysobacter*	*Gammaproteobacteria*	*Xanthomonadaceae*	338 (1.1%)	83 (0.2%)	6.00E-14
*Nitrospira*	*Nitrospira*	*Nitrospiraceae*	110 (<0.1%)	10 (<0.1%)	6.00E-14
*Nocardioides*	*Actinobacteria*	*Nocardioidaceae*	207 (0.7%)	42 (0.1%)	6.00E-14
*Paracoccus*	*Alphaproteobacteria*	*Rhodobacteraceae*	59 (<0.1%)	8 (<0.1%)	6.00E-14
*Pseudoxanthomonas*	*Gammaproteobacteria*	*Xanthomonadaceae*	212 (0.7%)	16 (<0.1%)	6.00E-14
*Roseomonas*	*Alphaproteobacteria*	*Acetobacteraceae*	109 (<0.1%)	399 (1.0%)	6.00E-14
*Subsaxibacter*	*Bacteroidetes*	*Flavobacteriaceae*	87 (<0.1%)	8 (<0.1%)	6.00E-14
*Thermomonas*	*Gammaproteobacteria*	*Xanthomonadaceae*	230 (0.8%)	18 (<0.1%)	6.00E-14

**FIGURE 4:**
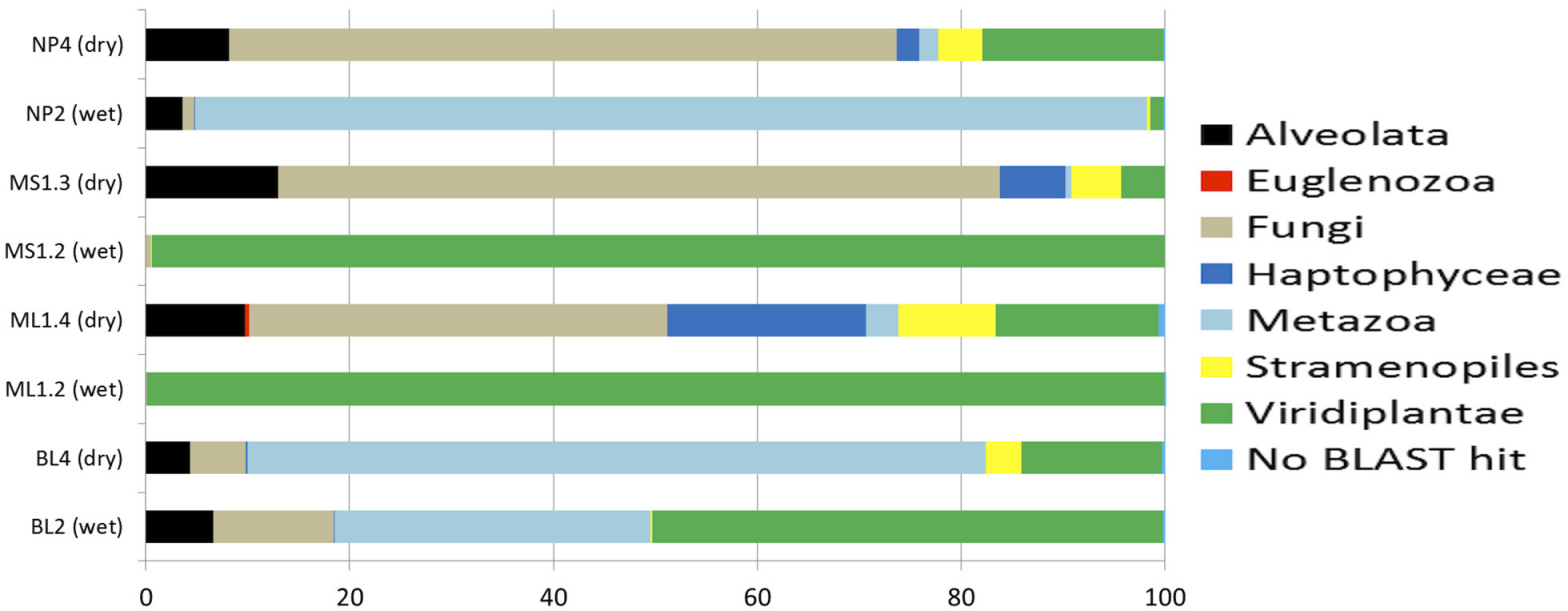
**Percentage composition of eukaryotic communities as based 18S rRNA-based amplicon pyrosequencing.** The total number of sequences for each sample: NP4, 2556; NP2, 9623; MS1-3, 1058; MS1-2, 9060; ML1-4, 2958; ML1-2, 14642; BL4, 5417; BL2, 2101.

For Eukarya (**Figure 4**), obvious differences included a fungal dominance in arid soils (~41–70%) with the exception of BL (~5.5%) and both the Haptophyceae (dominant signatures related to *Imantonia*, *Cruciplacolithus*, and *Reticulosphaera*) and Stramenopiles (dominant signatures being *Desmarestia*, *Paraphysomonas*, and *Chrysococcus*) being highly represented in arid soils (~0.1–20% and ~3.5–9.5%, respectively) as opposed to wet soils (~0.0–0.1% and ~0.0–0.4%, respectively). Another obvious trend was *Spathidium* (of the Alveolata) being highly represented in arid soils (1.6–8.6% of total community), but rare (0.04%) to undetected in wetted soils. Viridiplantae (mainly *Streptophyta* signatures, as reflecting 16S rRNA results) were more common in wet soils, ~50–99% for BL, ML, and MS (with the exception of NP, at 1.2%), with the number of sequences detected in arid soils being considerably lower (i.e., ~4–16% for BL, ML and MS and ~18% for NP).

## DISCUSSION

During the summer months hyporheic soils surrounding ephemeral DV streams and lake edges are hotspots of biological activity (Runkel et al., 1998; McKnight et al., 1999, 2007; Gooseff et al., 2003; Niederberger et al., 2012), and are hypothesized to be an important source of carbon and nitrogen to the surrounding soils (Hopkins et al., 2006a,b; Barrett et al., 2007; Novis et al., 2007). Although the significance of these transiently wet sites is acknowledged, there is limited information on the microbial communities inhabiting these soils. Our previous research has identified microbial assemblages in these wet soils that have the potential for N_2_ fixation with high rates of N_2_ fixation that may in-part be linked to sulfate reduction (Niederberger et al., 2012). Stanish et al. (2013) also recently reported in-depth analyses of microbial community structure of mats located in DV streams, the results of which showed significant relationships between diatom and bacterial communities and indicated species interactions between specific taxa. Moreover, results suggested that stream flow regime may influence the overall community structure with a lack of relationship between heterotrophic microbial diversity and nutrient concentrations (Stanish et al., 2013). It therefore seems that both species interactions and niche specific processes have an influence in defining community structure in DV microbial mats.

Our studies focused instead on wet soils at the edge of and adjacent to DV stream, lakes, and ponds (the hyporheic zone). Our transects, with sites sampled from the origin in wet soils and extending to arid soils varied in moisture content, with moisture contents at the farthest “point 4” sampling site being in the range typically reported for archetypal DV soils, i.e., ~<5% (Niederberger et al., 2008; Cary et al., 2010; Lee et al., 2012; Tiao et al., 2012). Arid soils were highly alkaline (~9.0) as expected in the Mier’s Valley locale (Lee et al., 2012), with values decreasing along the moisture gradient to near neutral levels at the leached wet origin soils. Due to the presence of cyanobacterial crust communities, wetter sites contained elevated chlorophyll *a* concentrations relative to arid soils.

Various studies have attempted to link physicochemical properties with soil microbial ecology in the DV soils (Virginia and Wall, 1999; Niederberger et al., 2008; Soo et al., 2009; Smith et al., 2010; Zeglin et al., 2011; Lee et al., 2012; Stanish et al., 2013). Water obviously drives biomass formation in DV soils as evidenced by large amounts of biofilm/biomass presence at lake edges; however, water may not be the primary driver of the presence of all organisms in DV soil habitats. For example, water does not appear to be the limiting factor for nematode abundance in DV soil habitats (Virginia and Wall, 1999) and water content is a poor indicator of cyanobacteria distribution in DV soils with other variables such as soil elemental composition or conductivity hypothesized to define habitat suitability (Wood et al., 2008). More recently, Lee et al. (2012) examined microbial communities from four geographically distinct DV soils and report significantly different communities between sites that were correlated to salt content, altitude and Cu^2+^. Smith et al. (2010) have also noted high spatial variability of bacterial communities in DV soils; however in their study, microbial signatures were correlated with soil moisture and pH (ϱ = 0.564). In a related study describing communities in DV hyporheic zones water content was strongly related to community composition with microbial diversity related to soil conductivity (Zeglin et al., 2011). Conductivity has also recently been shown to influence cyanobacterial diversity in microbial mats associated with streams in the DV (Stanish et al., 2013). Analogous to these collective studies, our work also suggests that water drives bacterial community composition as evidenced by bacterial T-RFLP fingerprints of drier sites clustering together within PCA ordinations. Weak correlations (ϱ = 0.214) were identified for bacterial community fingerprints that were most closely related to pH, NH_4_ and the distance from the water source [as previously postulated (Niederberger et al., 2012)] which could also be perceived as directly relating to percent moisture content. As previously hypothesized for nematode distribution (Virginia and Wall, 1999), moisture was not a strong driver of Eukarya presence in this study, with no similarity observed between wet or dry samples on PCA ordinations and only weak correlations to distance from source, % moisture and pH (ϱ = 0.202). Collectively, these studies suggest that a complex set of abiotic factors define habitat suitability for microbial communities in DV soils that currently remain unresolved.

Microbial communities in the arid DV soils have recently been shown to be highly localized with structurally and phlyogenetically distinct populations existing in geographically disparate sites, suggesting that communities maybe endemic to each DV location (Lee et al., 2012). This is in contrast to the hypothesis that the intense DV winds might distribute microbes throughout the valleys. In fact, high-throughput rRNA sequencing revealed that microbial signatures in aerosols and nearby soils were dominated by different phyla (Bottos et al., 2014) hinting that aeolian-based movement of soils and the associated communities among valleys may be limited and therefore local physicochemical factors will have a major role in shaping community structure and colonization of DV soils (Lee et al., 2012). This insight is also reflected in PCA ordinations of bacterial communities in wetter sites in our study (>5% moisture content), as sites located in Miers Valley (i.e., stream and lake systems) clustered together and transects located in adjacent northern (NP and BL) and southern sites (Hidden Valley) formed another cluster. These observed differences may be due to local physicochemical factors selecting for these communities, e.g., the source of the water and the local mineralogy. Yet, in contrast to the findings by Lee et al. (2012) bacterial fingerprints from all arid sites clustered tightly together within PCA ordinations, indicating a commonality of community structure between these particular arid soils. This may however be attributable to the different geographical scales between the two studies, with Lee et al. (2012) analyzing samples from four geographically disparate Valleys as opposed to the more local samples described in this study. Further work is therefore required to illuminate the spatial distribution and possible biogeography of microbial communities between arid DV soils.

Wet soils were dominated by Cyanobacterial signatures with the exception of BL2 where Bacteroidetes comprised ~34% of the community as compared to ~10% for cyanobacteria. Bacteroidetes related sequences also made up a large proportion of the signatures detected in both the wet and dry soils (~7–55%) at the other transect sites. In a similar study (Stanish et al., 2013), Bacteroidetes were the most abundant heterotrophic taxa detected in DV microbial mats (including MS). Globally, Bacteroidetes are common members of microbial mats and are suited to these types of environments due to their ability to degrade various organic compounds (Stanish et al., 2013), and as witnessed in the RDP-based comparison, there was a notable significant enrichment of Flavobacterium-related signatures of the Bacteroidetes in the wet soils. In contrast to this finding, the *Gillisia* genera of the Bacteroidetes was more abundant in arid soils and was the most significant difference between the wet and arid soils (discussed in detail below). Our results also mirror those reported by Stanish et al. (2013) whereby, Alphaproteobacteria and/or Betaproteobacteria were also dominant members of the microbial communities inhabiting ephemerally wet DV soils. Although RDP-based comparative analyses did not indicate that these phyla differed significantly between collective wet and arid soil sequences, certain genera within these phyla were significantly higher in wet soils, including *Erythromicrobium* [a freshwater, obligately aerobic, anoxygenic photosynthetic bacterium (Yurkov et al., 1994)], *Amaricoccus* [an aerobic chemo-heterotroph (Maszenan et al., 1997)], *Brevundimonas* [a radiation resistant organism previously isolated in Antarctic soils (Dartnell et al., 2010)], and *Roseomonas* [a pink-pigmented organism with an oxidative metabolism (Zhang et al., 2008)].

As expected (Niederberger et al., 2012), wetter soils had higher cyanobacterial presence compared to dry soils. Due to the RDP classifier employing the Bergeys Manual-based cyanobacterial classification of family level groupings, GpI–XIII (Bolhuis and Stal, 2011), the cyanobacterial signatures are not classified any deeper. The vast proportion of cyanobacterial signatures for all sites were unclassified with the GpI group of the Family I phylogenetic clade dominating cyanobacterial signatures in both wet and arid soils. GpI signatures were generally higher in wet soils, ~2–12% of the total community (0.2–3% in arid soils). The GpIV group of cyanobacteria were the second most dominant cyanobacterial signature with higher numbers in arid soils of BL and ML (~0.1 and 6% respectively) as opposed to wet (0.04 and 3%), and similar concentrations in wet and arid soils for MS and NP (~2 and 0.2%, respectively). However, of most interest is that the fact that RDP-based comparisons of combined signatures indicate significantly higher GpI signatures in wet soils, with GpIV signatures significantly higher in drier soils; therefore, these two groups of organisms may be adapted to their respective contrasting soil biotopes. Significantly higher quantities of chloroplast signatures were also detected in the wet soils at ML and MS sites (**Table 2**) that were almost exclusively related to the *Streptophyta*. Members of this green algal group inhabit both Arctic and Antarctic environments and have been studied due to their exposure to stressful environmental conditions including desiccation, high irradiation and UV levels (Kaplan et al., 2013). The GpI and IV groups of cyanobacteria have also been amongst the most dominant signatures detected in other environments that are exposed to sporadic, ephemeral wetting events including coastal microbial mats (Bolhuis and Stal, 2011) with the GpI group being abundant in Arctic snow layers (Møller et al., 2013). Eukarya presence in the DV soils also reflected previous studies, with a fungal dominance in arid soils and Viridiplantae in wet soils (Fell et al., 2006; Ruisi et al., 2007; Cary et al., 2010). These results therefore suggest the presence of cyanobacterial and algal species that are adapted to both the transient availability of water and inherent stressful conditions in these cold DV soils.

Pointing et al. (2009) have reported low or absent cyanobacterial signatures in arid soils of the McKelvey Valley, with cyanobacterial signatures being absent at all arid sites in the molecular-based studies by Niederberger et al. (2008), Smith et al. (2010), and Lee et al. (2012). Yet, at least in one case this has been attributed to PCR bias via use of a non-specific primer (Lee et al., 2012). Collectively, a higher abundance of signatures from the phyla, Acidobacteria, Actinobacteria, *Deinococcus/Thermus*, Bacteroidetes, Firmicutes, Gemmatimonadetes, Nitrospira, and Planctomycetes were detected in arid soils as compared to wet soils with various genera of these phyla being significantly more abundant in arid soils (**Table 2**). The most significant of these at the genus level was the presence of *Gillisia* in combined arid soils (3.9%), but a complete absence in wet soils. Cultured and characterized representatives of the *Gillisia* genera are typically psychrophilic, chemoheterotrophic organisms isolated from Antarctica habitats; however, unexpectedly, these species have been isolated from wet biotopes including mats from Lake Fryxell (Van Trappen et al., 2004) and Antarctic maritime habitats (Bowman and Nichols, 2005). Various studies have proven that Actinobacteria and/or Acidobacteria are dominant members of arid DV soils (Smith et al., 2006; Niederberger et al., 2008; Pointing et al., 2009; Lee et al., 2012; Bottos et al., 2014) and that the *Deinococcus/Thermus* group maybe specific to low-productivity DV soils (Niederberger et al., 2008). Recently, the *Deinococcus* class of microorganisms have been shown to form a distinct distribution pattern in DV stream associated mats (Stanish et al., 2013) with higher abundances linked to their inherent high-UV tolerance. Radiation resistant signatures related to *Truepera* of the *Deinococcus/Thermus* group and Actinobacterial members of the Rubrobacteridae, e.g., *Rubrobacter* and *Patulibacter* were also detected in our study, and continue to be revealed as dominant members of arid DV biotopes (Carpenter et al., 2000; Aislabie et al., 2006; Smith et al., 2006; Shravage et al., 2007; Niederberger et al., 2008; Lee et al., 2012). Likewise, the eukaryote, *Spathidium* was notably higher in dry soils which can be attributed to their ability to survive periods of dryness via dormant resting stages (cysts; Foissner, 1974).

In contrast to archetypal arid DV desert soils, few studies have applied molecular-based techniques on microbial communities inhabiting wet/high productivity sites (Niederberger et al., 2008, 2012; Zeglin et al., 2011; Stanish et al., 2013). This research represents the first in-depth characterization of both bacterial and eukaryotic communities inhabiting wet DV soils adding to the knowledge of these environments. A large percentage of bacterial signatures also remain unidentified as is typically reported in studies utilizing rRNA-based molecular techniques to survey DV environments (Niederberger et al., 2008; Lee et al., 2012). However, it is important to recognize that due to inherent PCR-biases and the presence of dead cells and/or naked DNA, these analyses may not reflect the true diversity of DV communities. It is therefore suggested that future studies incorporate microscopy-based methods such as fluorescent *in situ* hybridization (FISH) to reveal the true diversity and spatial distribution of these microorganisms in DV soil biotopes. Although this work represents the first major in-depth sequencing effort of hyporheic DV soils uncovering both diversity and identify of microbial inhabitants, the long-term goal of elucidating the associated biological function and reasons for their biogeography remains unresolved.

## Conflict of Interest Statement

The authors declare that the research was conducted in the absence of any commercial or financial relationships that could be construed as a potential conflict of interest.
